# Clustering of Beijing genotype *Mycobacterium tuberculosis* isolates from the Mekong delta in Vietnam on the basis of variable number of tandem repeat versus restriction fragment length polymorphism typing

**DOI:** 10.1186/1471-2334-13-63

**Published:** 2013-02-02

**Authors:** Mai NT Huyen, Kristin Kremer, Nguyen TN Lan, Tran N Buu, Frank GJ Cobelens, Edine W Tiemersma, Petra de Haas, Dick van Soolingen

**Affiliations:** 1 Phạm Ngọc Thạch hospital, 120 Hung Vuong, district 5, Ho Chi Minh City, Viet Nam; 2 World Health Organization, Regional Office for Europe, Copenhagen, Denmark; 3 Tuberculosis Reference Laboratory, RIVM, PO Box 1, Bilthoven, 3720 BA, The Netherlands; 4 Centre for Infection and Immunity Amsterdam, Academic Medical Centre, Amsterdam, The Netherlands; 5 KNCV Tuberculosis Foundation, PO Box 146, The Hague, 2501 CC, The Netherlands; 6 Departments of Pulmonary Diseases and Medical Microbiology, Radboud University, PO box 9101, Nijmegen, 6500 HB, The Netherlands

## Abstract

**Background:**

In comparison to restriction fragment length polymorphism (RFLP) typing, variable number of tandem repeat (VNTR) typing is easier to perform, faster and yields results in a simple, numerical format. Therefore, this technique has gained recognition as the new international gold standard in typing of *Mycobacterium tuberculosis*. However, some reports indicated that VNTR typing may be less suitable for Beijing genotype isolates. We therefore compared the performance of internationally standardized RFLP and 24 loci VNTR typing to discriminate among 100 Beijing genotype isolates from the Southern Vietnam.

**Methods:**

Hundred Beijing genotype strains defined by spoligotyping were randomly selected and typed by RFLP and VNTR typing. The discriminatory power of VNTR and RFLP typing was compared using the Bionumerics software.

**Results:**

Among 95 Beijing strains available for analysis, 14 clusters were identified comprising 34 strains and 61 unique profiles in 24 loci VNTR typing ((Hunter Gaston Discrimination Index (HGDI = 0.994)). 13 clusters containing 31 strains and 64 unique patterns in RFLP typing (HGDI = 0.994) were found. Nine RFLP clusters were subdivided by VNTR typing and 12 VNTR clusters were split by RFLP. Five isolates (5%) revealing double alleles or no signal in two or more loci in VNTR typing could not be analyzed.

**Conclusions:**

Overall, 24 loci VNTR typing and RFLP typing had similar high-level of discrimination among 95 Beijing strains from Southern Vietnam. However, loci VNTR 154, VNTR 2461 and VNTR 3171 had hardly added any value to the level of discrimination.

## Background

The IS*6110* restriction fragment length polymorphism (RFLP) typing was previously considered the gold standard in the molecular epidemiology of tuberculosis [1]. Although this typing technique generally revealed a high level of discrimination among *Mycobacterium tuberculosis* isolates, it was considered complicated, technically demanding, and time consuming. In addition, a part of the strains contained too few copies of IS*6110* to enable a reliable typing. Variable number of tandem repeat (VNTR) typing is easier and faster to perform, and yields results in a numerical format. Therefore, this technique has become the new international typing method for *M. tuberculosis* since 2006 [2]. Several studies indicated that VNTR typing is as discriminative as RFLP typing and more suitable to type strains with few copies of IS*6110*[3,4]. However, doubt remained whether VNTR typing is as good as RFLP typing in discriminating Beijing genotype strains. As in Vietnam about 40% of the *M. tuberculosis* isolates are of this genotype, we in this study compared the performance of RFLP and internationally standardized 24 loci VNTR typing to discriminate among one hundred Beijing genotype isolates from the South of Vietnam.

## Methods

### Study population

In total 100 *M. tuberculosis* isolates of the Beijing genotype family were selected from a previous study on the dynamics of tuberculosis transmission in Vietnam. The study area consisted of three adjacent rural districts in Tiengiang Province, in the Mekong River Delta in Southern Vietnam. All patients were aged ≥15 years, resident in the study area and registered for treatment of smear-positive pulmonary tuberculosis (TB) between 1 January 2003 and 28 June 2007 at the participating District Tuberculosis Units, or at the provincial TB hospital and were eligible for inclusion into the study. Each eligible patient submitted two sputum samples for TB culture, drug susceptibility testing and genotyping and completed an interview form. The details of this study have been published previously [5].

### Ethical approval

Ethical clearance was obtained from the ethical health committee of the Ho Chi Minh City Council (reference number 1106/UBND-VX). All included patients provided written informed consent.

### *Mycobacterium tuberculosis* culture

Sputum specimens were kept refrigerated and transported to Pham Ngoc Thach Hospital in Ho Chi Minh City within 72 hrs after collection. They were decontaminated and liquefied using 1% N-acetylcysteine/2% NaOH, inoculated on modified Ogawa medium and incubated at 37°C. Cultures were examined for growth after 1, 2, 4, 6 and 8 weeks of incubation. Cultures with no growth after 8 weeks were considered negative. *M. tuberculosis* was identified using the niacin and the nitrate tests [6].

### DNA typing

Genomic DNA was extracted from positive cultures using an earlier described method [7]. IS*6110* RFLP typing and spoligotyping were performed according to the internationally standardized methods [1,8]. The VNTR typing was executed on the basis of 15 loci and 24 loci as described by Supply *et al*. [2].

### Random selection of one hundred Beijing genotype strains

Among 1,797 *M. tuberculosis* isolates that were successfully typed in RFLP and spoligotyping, 819 strains represented the Beijing genotype according to spoligo typing patterns. After the isolate numbers had been sorted in numerical order, every 8^th^ Beijing genotype was selected until a total of 100 isolates was reached.

### Data analysis

Gene Marker software, version 1.5 (Softgenetics, PA, USA) was used for analysis and automated allele calling of the VNTR patterns. The Bionumerics software, version 3.0 (Applied Maths, Sint-Martens Latem, Belgium) was used for the analysis and comparison of IS*6110* RFLP and VNTR typing patterns.

The Hunter Gaston Discrimination Index (HGDI) was used to analyse the discrimination power of VNTR and RFLP typing results [9]:

(1) $$D=1-\frac{1}{n\left(n-1\right)}\sum_{j=1}^{S}\mathrm{nj}\left(\mathrm{nj}-1\right)$$

where n is the total number of strains in the sample population, s is the total number of types described, and nj is the number of strains belonging to the j^th^ type. This equation is derived as follows: the probability that a single strain sampled at random will belong to the j^th^ group is nj/ n and the probability that two strains sampled consecutively will belong to that group is nj(nj - 1)/ n(n - 1).

### Definitions

Beijing lineage (genotype) strains were defined as strains having at least three of the nine spacers 35 to 43 and lacking spacers 1–34 based on the 43 spacer spoligo patterns [8,10]. If a strain missed all spacers 1–34 and also one or a few of the spacers 35–43, the Beijing strains was considered to represent the Atypical branch of the Beijing genotype lineage [11].

Two strains were defined as a cluster if they had identical RFLP patterns or identical VNTR profiles (Bionumerics), or if VNTR types differed by no more than a single locus [2].

## Results

In the period January 2003 to June 2007, a total of 2,664 *M. tuberculosis* strains were isolated from eligible patients, of which 1,795 were successfully typed in RFLP and spoligo typing. Of these, 819 (45.6%) were of the Beijing genotype based on the spoligo patterns; the remaining 976 (54.4%) were of other genotypes. Among the 819 Beijing genotype strains, 41 (5.0%) most likely belonged to Atypical Beijing lineage, as they missed one or more spacers of the characteristic 9 spacer signature.

Of the 819 Beijing strains, 353 (43.1%) were isolated from patients in the Cailay district; 221 strains (27%) isolated from patients in the Caibe district, and the remaining 245 strains (29.9%) from patients in the Chauthanh district. Regarding the gender of patients, 592/819 (72.3%) of the strains were isolated from male patients, the remaining 227 (27.7%) from females.

From these 819 Beijing genotype strains, 100 strains were randomly selected as described above, of which 5% were most likely Atypical Beijing strains because they missed one or two spacers of the characteristic nine spacer panel 35–43.

Among the 100 selected Beijing genotype strains, 38 (38%) were isolated in the Cailay district, 32 (32%) in the Caibe district and 30 (30%) in the Chauthanh district. The gender distribution was 71% males and 29% female patients. Therefore, the distribution of sex, districts, age (data not shown) in the representative Beijing strains was similar to that in the total collection of 819 Beijing strains (P>0.05).

**Figure 1:**
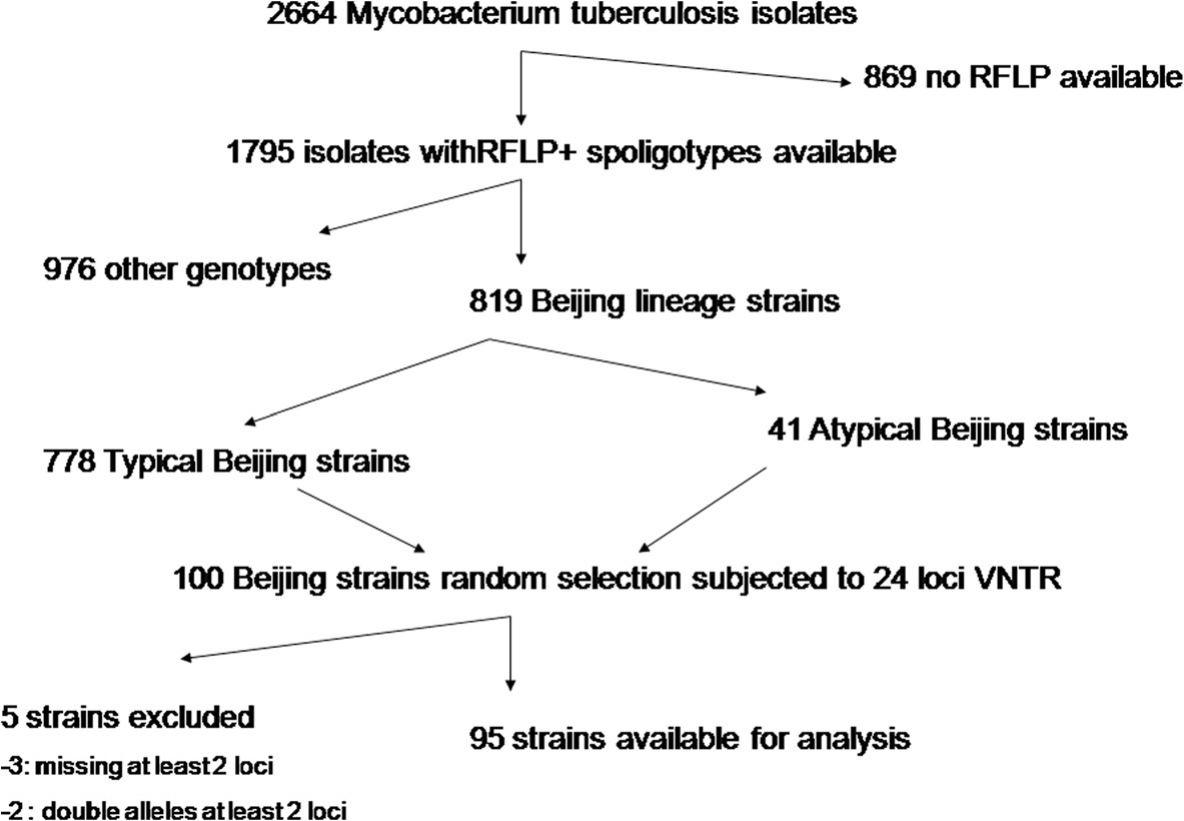
Study flow chart.

Among the 100 Beijing isolates that were subjected to 24 loci VNTR typing, 95 yielded results suitable for analysis (including 88 that yielded results for all 24 loci and 7 with double alleles or that missed one locus only), the remaining 5 strains were excluded because ambiguous PCR results were obtained or double alleles were observed in at least two loci. Among 3/5 isolates at least two loci could not be amplified and two isolates had double alleles in two or more loci (Figure 1).

**Figure 2:**
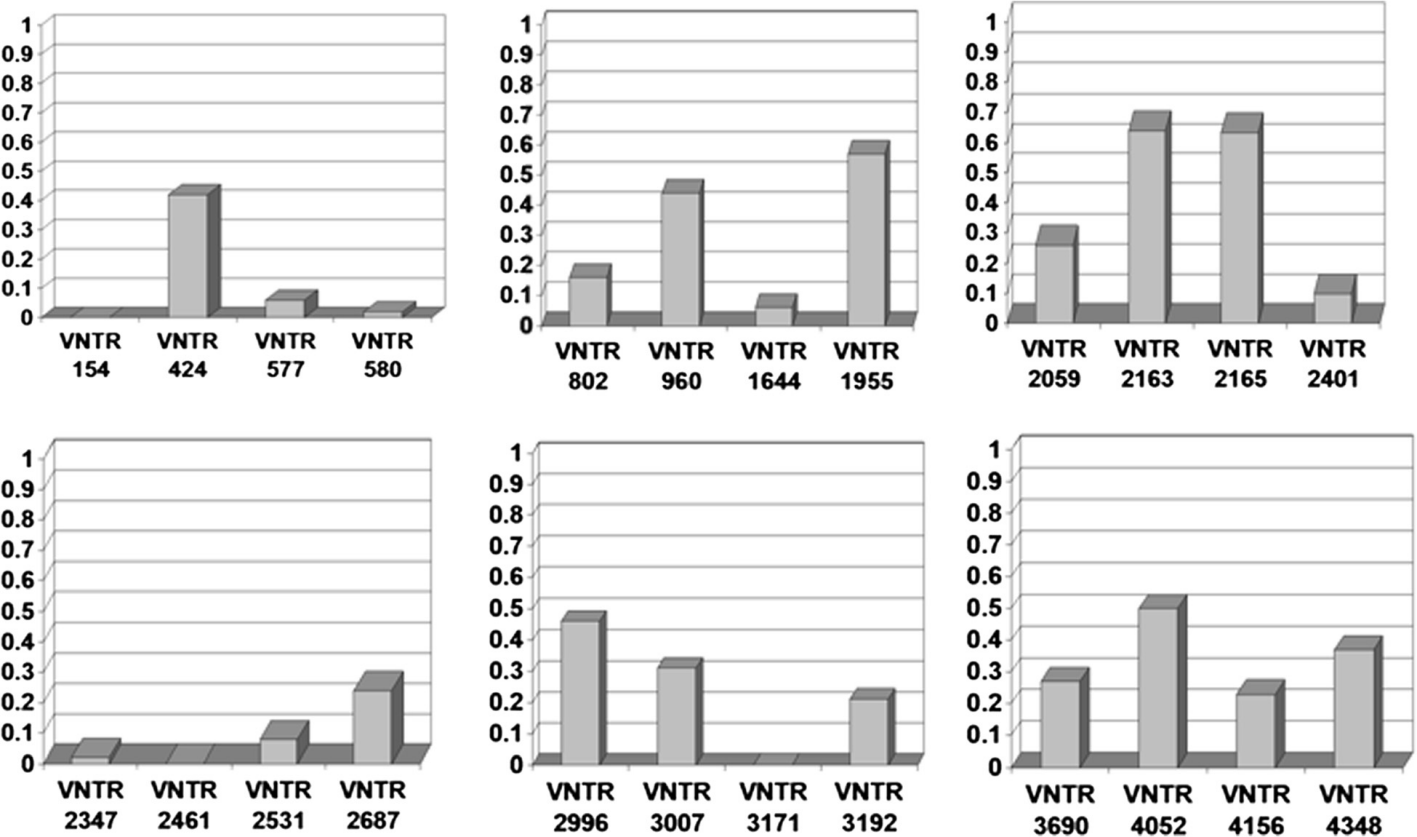
**Hunter Gaston discrimination index of 24 VNTR loci among 95 Beijing strains.** Y axis: Hunter Gaston discrimination index.

Of the 24 loci analyzed, VNTR 154, VNTR 2461 and VNTR 3171 had no discrimination power and hence, the HGDI was zero. Loci VNTR 2347, VNTR 580, VNTR1644, VNTR 0577, VNTR 2531, VNTR 2401 and VNTR 802 had a HGDI of less than 0.2. The loci having a HGDI of more than 0.4 were VNTR 424, VNTR 960, VNTR 2996, VNTR 4052, VNTR1955, VNTR 2165 and VNTR 2163b. Locus VNTR 2163b had the highest allelic diversity, with a HGDI of 0.64 (Figure 2 and Table 1).

**Table 1:** Hunter Gaston discrimination index (HGDI) values obtained for each locus in the present study compared to two other studies

**VNTR locus**	**Present study**	**Kremer*et al.***[4]	**Alonso*et al.***[12]
154	0.00		0.23
2461	0.00	0.00	
3171	0.00		
2347	0.02		
580	0.02	0.019	0.21
1644	0.06	0.058	0.455
577	0.06	0.165	0.63
2531	0.08		0.655
2401	0.10		0.65
802	0.16	0.196	0.73
3192	0.21		0.36
4156	0.23		0.53
2687	0.24		0.06
2059	0.26		0.16
3690	0.27		0.64
3007	0.31		0.13
4348	0.37	0.32	0.09
424	0.42		0.66
960	0.44	0.377	0.685
2996	0.46	0.2	0.46
4052	0.50	0.299	0.8
1955	0.57		0.65
2165	0.635	0.201	0.61
2163b	0.64	0.618	0.78

In the 24 loci VNTR typing of the remaining 95 isolates, 14 clusters of isolates were found; one cluster of four isolates, four clusters of three isolates and nine clusters of two strains. Sixty-one isolates revealed a unique VNTR pattern. The HGDI amounted to 0.994. Twelve of the VNTR clusters were subdivided in RFLP typing.

In RFLP typing, 13 clusters of isolates were found, comprising in total of 31 strains. There were ten clusters of two, two clusters of three and one cluster of five isolates. Sixty-four isolates had a unique RFLP pattern (HGDI = 0.994). Nine RFLP clusters were subdivided in VNTR typing.

## Discussion

From 1993 to 2006, RFLP typing was considered the gold standard in typing of *M. tuberculosis* isolates, especially for strains harboring multiple IS*6110* copies, like the ones of the Beijing genotype family. However, this typing method is technically demanding and time consuming [4]. Furthermore, the discriminatory power of RFLP typing among strains with a low number of IS*6110* copies (<= 5 copies) is very poor [3]. Therefore, in recent years VNTR typing has increasingly been explored in the molecular epidemiology worldwide and with the proposal on international standardization of this technique in 2006 it has in fact become the new gold standard [2]. VNTR typing introduced major advantages in typing in comparison to RFLP typing, such as its ease in use, its suitability for standardization, and that the results that are displayed in numbers can be analyzed easily and exchanged efficiently between laboratories. Moreover, the turnaround time of VNTR typing is much shorter than that of RFLP typing, because it is PCR-based and only a little amount of mycobacterial DNA is required.

Many researchers have carried out comparisons between RFLP and 12 or 15 loci VNTR typing methods for discriminating *M. tuberculosis* isolates [3,4,12]. Their findings showed that the discriminative power of 12 loci VNTR was lower than that of 15 loci VNTR (with HGDI of 0.978-0.995) [12] and 15 loci VNTR has high level of discrimination with HGDI 0.990-0.995 [4,12], but this was still lower than that of RFLP typing (0.998) [4]. However, Supply *et al*. [2] proposed to apply 24 instead of 15 loci in VNTR typing, and this improved the level of discrimination significantly.

In our study, we compared the performance of 15 and 24 loci VNTR typing and RFLP typing using 95 Beijing strains and we found that the discrimination index (HGDI) of 15 loci VNTR was the lowest (0.992), followed by both RFLP typing and 24 loci VNTR typing (0.994). However, the differences observed were small. The HGDI of some loci (VNTR 154, VNTR 2461,VNTR 3171) were low in our study, which means that these loci are less useful in discriminating Beijing strains in the South of Vietnam and presumably elsewhere. The HGDI of VNTR 2461, VNTR 577, VNTR 2163b, VNTR 580, VNTR 802, VNTR 960, VNTR 1644 and VNTR 4348, were similar to that observed in a previous study in Hong Kong [4] (Table 1), whereas the HGDI of VNTR 2996, VNTR 4052, and VNTR 2165 were significantly higher than the ones in that study [4] (Table 1), for unknown reasons. It may be that because BCG vaccination has been introduced much earlier in Hong Kong than in Vietnam, the ongoing selection of particular strains of the Beijing lineage [11] may be more advanced in the former than in the latter area and the mentioned loci may have a different level of discriminative power among the circulating strains in both areas.

Our study further found the HGDI of VNTR 1955, 2163b and 2165 to be very high (>0.50) and the best differentiation, similar to two previous studies [4,12], was obtained with VNTR 2163b (Table 1 and Figure 2).

Some of the HGDI of individual loci in our study were significantly different to the ones found in the study of Alonso *et al.*[12] (Table 1), because we performed VNTR typing of exclusively Beijing strains, whereas Alonso *et al.*[12] carried out VNTR typing on a strain collection consisting of 32% LAM, 28% Haarlem and only 2% Beijing strains.

A disadvantage of VNTR typing encountered in this study was that six strains revealed double alleles in a single locus, and two strains even in two and more than two loci. It is not clear whether the latter observation was associated with a mixed infection [13]. However, the revealed genomic instability in particular loci decreases the utility of VNTR typing significantly, as this hampers a reliable interpretation. Also in RFLP typing transposition of IS*6110* sometimes interfered with a reliable interpretation, but such a genetic turn-over was observed less frequently [14]. However, we cannot exclude the possibility that these multiple alleles may reflect important phenomena in the epidemiology of TB currently unknown, and these observations, although technically demanding, may be associated with the ongoing adaptation of *M. tuberculosis* to the current TB control measures.

A major limitation of this study was that we did not have epidemiological information available to verify the transmission links indicated by both typing methods. It was therefore, not possible to ascertain the validity of epidemiological links indicated.

## Conclusions

In comparison to 15 loci VNTR, RFLP typing and 24 loci VNTR typing revealed the highest level of discrimination among 95 isolates of the Beijing genotype from Southern Vietnam. For this and other practical reasons, the last method is preferred in investigation on transmission of Beijing strains in Vietnam. This method is in principle also useful in screening for possible mixed infections, after which positive findings (more than two loci with double alleles) would be confirmed with other methods.

## Competing interests

The authors declare that they have no competing interests.

## Authors’ contributions

MH was involved in data collection, analysis and in writing the manuscript. KK, NL, ET, FC, PH and DS were involved in the conception of the study and in writing the manuscript. TB was involved in data collection and in writing the manuscript. All authors read and approved the final manuscript.

## Pre-publication history

The pre-publication history for this paper can be accessed here:

http://www.biomedcentral.com/1471-2334/13/63/prepub
